# Characterisation of CD4+ T-cell subtypes using single cell RNA sequencing and the impact of cell number and sequencing depth

**DOI:** 10.1038/s41598-020-76972-9

**Published:** 2020-11-13

**Authors:** James Ding, Samantha L. Smith, Gisela Orozco, Anne Barton, Steve Eyre, Paul Martin

**Affiliations:** 1 Centre for Genetics and Genomics Versus Arthritis, Centre for Musculoskeletal Research, Manchester Academic Health Science Centre, The University of Manchester, AV Hill Building, Oxford Road, Manchester, M13 9PT UK; 2 The Lydia Becker Institute of Immunology and Inflammation, Faculty of Biology, Medicine and Health, University of Manchester, Manchester, UK; 3 NIHR Manchester Biomedical Research Centre, Manchester University NHS Foundation Trust, Manchester Academic Health Science Centre, Manchester, UK

**Keywords:** Autoimmunity, Cell biology, Immunology

## Abstract

CD4+ T-cells represent a heterogeneous collection of specialised sub-types and are a key cell type in the pathogenesis of many diseases due to their role in the adaptive immune system. By investigating CD4+ T-cells at the single cell level, using RNA sequencing (scRNA-seq), there is the potential to identify specific cell states driving disease or treatment response. However, the impact of sequencing depth and cell numbers, two important factors in scRNA-seq, has not been determined for a complex cell population such as CD4+ T-cells. We therefore generated a high depth, high cell number dataset to determine the effect of reduced sequencing depth and cell number on the ability to accurately identify CD4+ T-cell subtypes. Furthermore, we investigated T-cell signatures under resting and stimulated conditions to assess cluster specific effects of stimulation. We found that firstly, cell number has a much more profound effect than sequencing depth on the ability to classify cells; secondly, this effect is greater when cells are unstimulated and finally, resting and stimulated samples can be combined to leverage additional power whilst still allowing differences between samples to be observed. While based on one individual, these results could inform future scRNA-seq studies to ensure the most efficient experimental design.

## Introduction

Whilst genetics studies have been successful in identifying single nucleotide polymorphisms (SNPs) associated with common complex disease susceptibility, mortality and outcome^1–3^, they have had limited impact for predicting treatment response and there is currently great interest in discovering biomarkers which can predict if a patient will respond to a given therapy^4^. Studies to date have focused on bulk assays in serum or whole blood, including ELISAs and RNA-seq, and as such cannot fully explore the heterogeneity present in a sample.

CD4+ T-cells are established as an important cell type in the pathogenesis of common, complex autoimmune disease, including rheumatoid arthritis (RA), Crohn’s disease, multiple sclerosis and systemic lupus erythematosus (SLE)^5^. This is mostly due to their involvement in adaptive immunity but more recently genetic evidence, pathway analyses and the success of abatacept (CTLA4 antagonist) and infliximab (TNFα antagonist) in the treatment of RA and Crohn’s disease, for example, have added further evidence^3,6–8^. However, it is clear that within CD4+ T-cells, many different, functionally distinct cell types exist such as naïve, helper and regulatory T-cells and these cell types can be divided further still. For example, T helper cells can be classified into Th1, Th2, Th17 or T_FH_ cells based on their expression of certain transcription factors and cytokines^9^.

Single cell genomic approaches, such as mass cytometry by time of flight (CyTOF) and single cell RNA-seq (scRNA-seq), have the potential to fully explore this heterogeneity by independently assaying individual cells. This can help disentangle the population substructure and identify differences, such as rare populations or changes in sub-type frequency, between two conditions. Indeed, using CyTOF, Rao et al*.* identified a subset of T-helper cell, characterised by high PD-1 expression, which were expanded in the synovium of seropositive RA patients compared to seronegative RA patients^10^. This approach validates the use of single cell genomics in complex disease research but requires the development of a limited panel of 30–40 markers, which only allows the testing of specific hypotheses.

By contrast, scRNA-seq employs an unbiased, hypothesis-free approach to measure the RNA species present in each cell. As such, it has been widely used to characterise heterogeneous cell types, explore cell differentiation and identify cell sub-types involved in health and disease. Furthermore, the development of droplet-based systems, such as Drop-Seq^11^ or the 10x Genomics Chromium Controller^12^, allows researchers to study thousands of cells, overcoming the limitation of cell number in lower throughput microfluidic or plate-based techniques. This allows the accurate profiling of more complex cell populations in a high throughput, cost-effective manner.

Two key considerations for designing scRNA-seq experiments are read depth and cell number. Although it has been shown that for the Fluidigm microfluidics platform 50,000 reads per cell were sufficient to classify broad cell types, between 500,000 and one million reads per cell were required to detect a fuller range of expressed genes and quantify subtle expression changes^13^. Therefore, while increases in both cell number and read depth will provide more power to classify cell sub-types and identify rare populations, cost implications result in a compromise based on experimental objectives. Current recommendations for droplet-based systems are in the region of 20,000–50,000 reads per cell, partly because these methods rely on a 3′ mRNA-seq assay as opposed to the full-length assay often employed by other non-droplet based techniques. Despite this recommendation, it is still advisable to adjust this depth depending on cell type and experimental requirements, as the coarse characterisation of diverse populations is achievable at lower depths, while the exploration of biological process associated with more subtle changes will require deeper sequencing depth^14^. When considering cell number, there are no accepted recommendations as this is highly dependent on experimental requirements and sample heterogeneity. The more heterogeneous the sample is the more cells will be required to capture the true variability over technical noise. For example, in an analysis of a dataset on approximately 2700 peripheral blood mononuclear cells (PMBCs), it was possible to easily identify eight major cell populations, including CD4+ T-cells, CD8+ T-cells, B-cells and monocytes. However, by increasing the cell number to approximately 68,000 cells it was possible to further resolve the CD4+ T-cells into groups representing naïve, memory and regulatory CD4+ T-cells^12^. Although new modelling approaches for normalisation^15^ are able to resolve some subtypes with fewer cells when compared to the standard workflow (https://satijalab.org/seurat/v3.1/sctransform_vignette.html).

Despite the importance of CD4+ T-cells in several diseases, particularly RA, there has been limited research into optimising experimental considerations using droplet-based scRNA-seq technologies. It is therefore unclear on whether scRNA-seq is able to characterise the heterogeneity of highly similar, but functionally distinct, CD4+ T-cells and the best experimental strategy to achieve this. The aim of the current study was to determine the optimal future study design for CD4+ T-cells. Specifically we investigated the impact of sequencing read depth and cell numbers both in terms of the accuracy and sensitivity to detect CD4+ T-cell sub-types. Furthermore, we explored the effect of T-cell receptor (TCR) stimulation to determine the potential of scRNA-seq to identify T-cell signatures under resting and stimulated conditions, for example, in order to compare patients with different disease activities within the same group in studies of treatment response.

## Results

**Figure 1:**
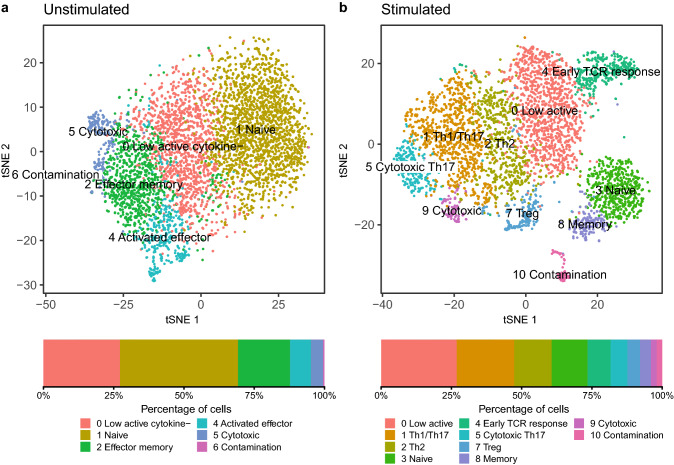
tSNE and cell frequency plots for (**a**) unstimulated and (**b**) stimulated samples. Clusters (top) and frequency plots (bottom) are coloured according to Seurat cluster identity and labelled according to inferred sub-type (Supplementary Figs. 2–7).

We recovered 5586 unstimulated cells and 4621 stimulated cells, corresponding to a read depth of 260,849 and 333,333 mean reads per cell for unstimulated and stimulated samples respectively. Both samples reached a sequencing saturation rate of 96.5%. However, while this resulted in a similar total number of detected genes, albeit at different read depths, the stimulated sample showed > 30% more median genes per cell (1443 vs 1090, Table 1), likely due to transcriptional activation upon stimulation. Furthermore, the unstimulated sample showed almost double the percentage of poor quality cells or suspected multiplets (8.8% vs 4.8%), compared to the stimulated sample. Graph based clustering of cells resulted in six clusters for the unstimulated sample and eleven clusters for the stimulated sample (Fig. 1).

**Table 1:** Sequencing metrics for the high depth datasets.

Sample	Cells recovered	Recovery rate	Cells passing QC	Sequencing saturation	Mean reads per cell	Total genes detected	Median number of genes
Unstimulated	5586	53.7%	5092	96.5%	260,849	19,508	1090
Stimulated	4621	44.6%	4400	96.5%	333,333	19,425	1443

### Cell quality is independent of read depth

**Figure 2:**
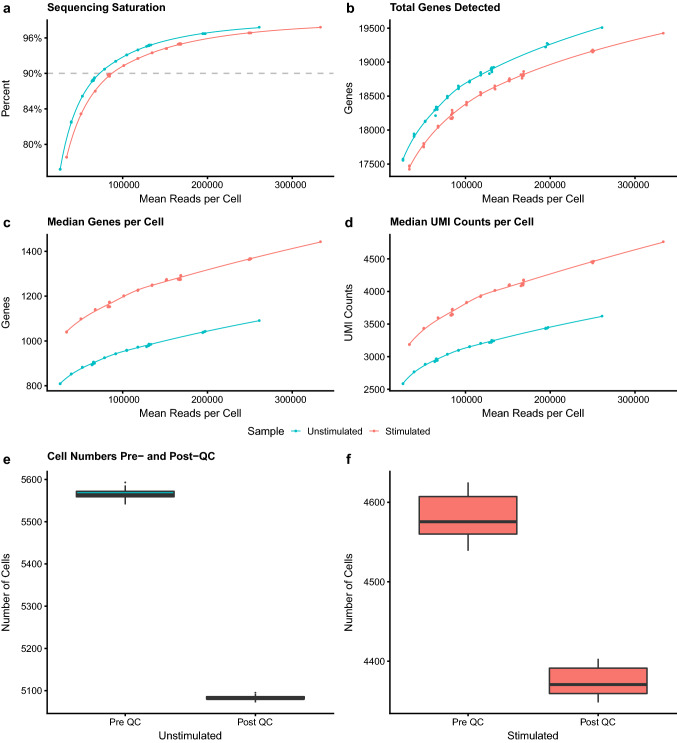
Sequencing metrics. Panels a-d show the effect of read depth on (**a**) sequencing saturation, (**b**) total genes detected, (**c**) median genes per cell and (**d**) median unique molecular identifier (UMI) counts per cell. Panels e and f show cell numbers pre and post quality control for (**e**) unstimulated and (**f**) stimulated samples. Unstimulated datasets are shown in blue and stimulated datasets in red.

Subsampling of read depth showed that, as expected, sequencing saturation quickly rose to around 90% where it started to plateau (Fig. 2a). This corresponded to a read depth of around 70–90 thousand reads per cell. The stimulated sample showed a higher total number of genes and displayed between 200 and 350 more median genes per cell and 600–800 more median UMI counts per cell, suggesting that, as expected, the stimulated sample is more transcriptionally active (Fig. 2b–d). Interestingly, both pre- and post-QC cell numbers remained fairly stable across all read depths for both unstimulated and stimulated samples (Fig. 2e,f), even after quality control, suggesting the ability to identify poor quality cells is independent of read depth or number of genes detected.

### Unstimulated cell clustering is more affected by sequence read depth

**Figure 3:**
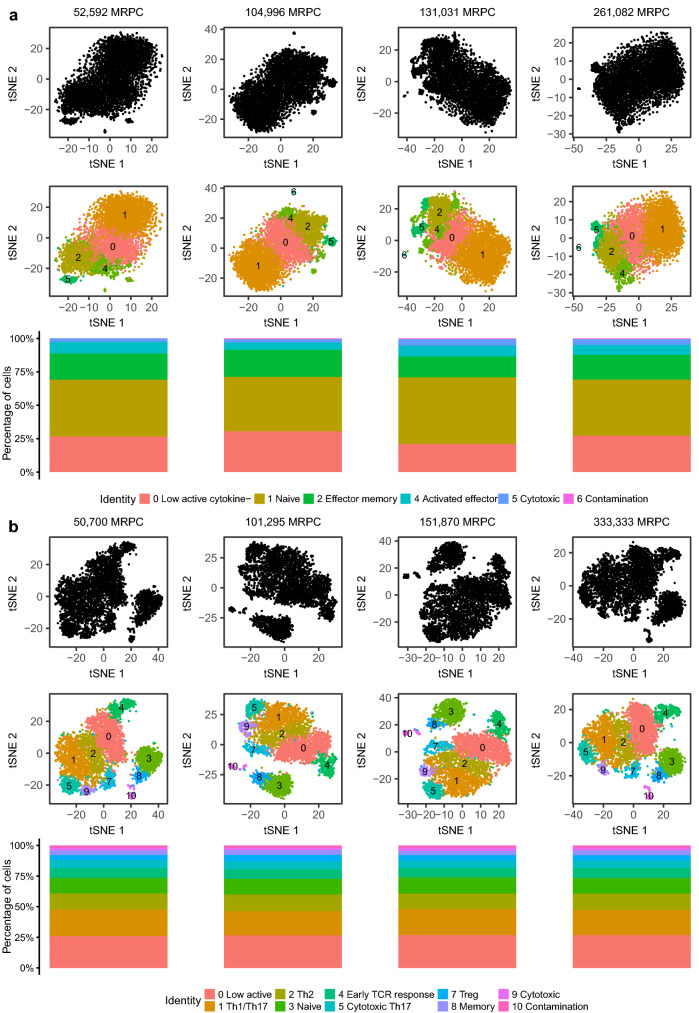
tSNE and cell frequency plots at selected read depths for (**a**) unstimulated datasets and (**b**) stimulated datasets. Top rows (black) show overall sample structure, middle rows show cluster identity and bottom rows show cluster frequency. Middle and bottom rows are coloured by cluster identity.

While these metrics show that there is more to gain from increased sequencing over 300,000 reads per cell, it is less clear whether these gains are biologically meaningful and/or effect the potential to identify T-cell subsets. To investigate this, clusters identified by graph based clustering of subsampled datasets were compared to the high depth sequencing datasets. Visual inspection of unstimulated and stimulated tSNE plots (Fig. 3) confirmed that the stimulated sample produced better cluster separation across a range of read depths. Furthermore, the stimulated sample produced more consistent results, identifying all clusters present in the high sequencing depth sample, even at ~ 50,000 reads per cell.

**Figure 4:**
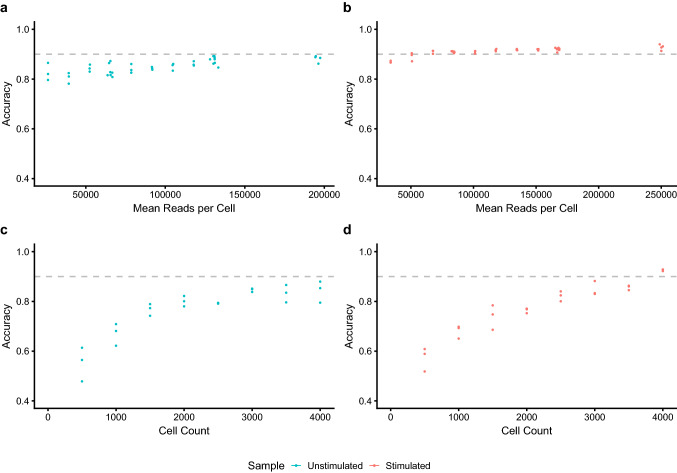
Cell type classification accuracy for (**a**) unstimulated and (**b**) stimulated datasets. Panels a & b show the effect of read depth of classification accuracy. Panels c & d show the effect of cell number on classification accuracy. Dashed lines indicates a 90% accuracy level.

**Figure 5:**
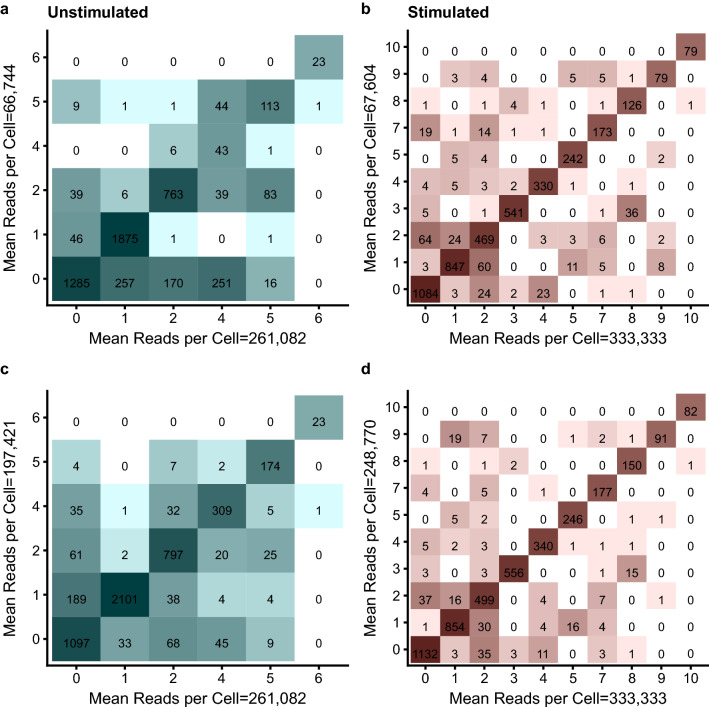
Confusion matrices comparing cell type classification between full sequencing depth and low/high subsampled depths. Tiles are shaded and numbered according to the number of cells classified and diagonal tiles represent concordant cell type classification between depths. Unstimulated sample (**a**) 261,082 versus 66,744 reads per cell and (**c**) 261,082 versus 197,421 reads per cell. Stimulated sample (**b**) 333,333 versus 67,604 reads per cell and (**d**) 333,333 versus 248,770 reads per cell.

To formally assess the accuracy of the clustering in respect to cell identity, cluster identity was confirmed at the single cell level. As expected from the tSNE plots, accuracy was lower for the unstimulated sample across the range of read depths tested (Fig. 4a), demonstrating 82.6% accuracy at a depth of approximately 65,000 reads per cell, compared to 91.3% at similar depth for the stimulated sample (Fig. 4b). Furthermore, the unstimulated sample achieved only an 89.2% maximum accuracy compared to 94% for the stimulated sample. This is also evident from the confusion matrices (Fig. 5a,c), where, with some exceptions, relatively few cells were misclassified for the stimulated sample and a median sensitivity of 92% and specificity of 99.4% was achieved.

By contrast, the unstimulated sample showed a much higher level of misclassification across all read depths compared to the stimulated sample (Fig. 5). Furthermore, there was greater variation between replicates at lower read depths compared to higher read depths. At 67,000 reads per cell, misclassification caused cells belonging to multiple clusters at high depth to be assigned to cluster 0 (Low active cytokine) and vice versa. Additionally cluster 4 (Activated effector) in the high depth sample was almost entirely missing, with the exception of 43 cells (sensitivity = 11.4%, specificity = 99.9%). Using an increased read depth of 197,000 reads per cell, a similar pattern of misclassification was observed, although cluster 4 did show improved classification (sensitivity = 89.9%, specificity = 97%).

### Cell number is more important than read depth

In contrast to the relatively small effect of read depth subsampling, cell number had a much more profound effect (Fig. 4c,d) on both unstimulated and stimulated samples. For the unstimulated sample sequenced at high depth, accuracy quickly dropped to below 80% for 2500 cells and less than 60% for 500 cells. Similarly, accuracy also suffered due to lower cell numbers for the stimulated sample, reducing to less than 90% for 3500 cells, less than 70% for 1000 cells and less than 60% for 500 cells. Furthermore between replicate variability was also higher for cell subsampling compared to read depth subsampling and this variability, in general, increased at lower cell numbers.

**Figure 6:**
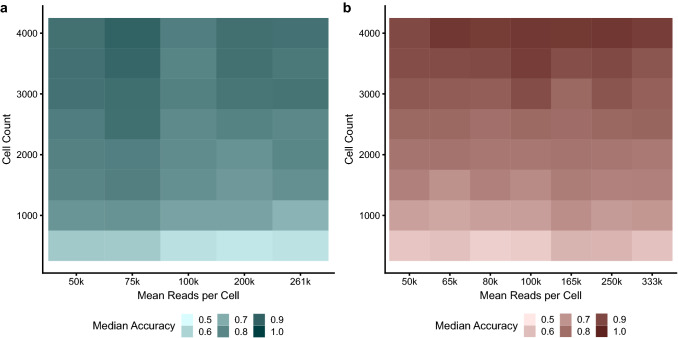
Heat map showing median accuracy as a function of read depth and cell number. (**a**) Unstimulated sample. (**b**) Stimulated sample. Tiles are shaded according to median accuracy between replicates.

To ensure that the decrease in accuracy was not affected by read depth, further cell number subsampling was performed using selected read depths (Fig. 6). This confirmed that the decrease in accuracy was not dependant on read depth, but was a result of decreased cell numbers.

### Cell type is more pronounced in stimulated cells

Both samples each showed a small cluster, present at 0.5% and 1.8%, for unstimulated (cluster 6) and stimulated (cluster 10) samples respectively, which was clearly separate from all others (Fig. 1). These clusters exhibited no expression of CD4 in either sample and no expression of CD3E and CD3G for unstimulated and stimulated samples respectively. Furthermore, less than 13% of these cells showed any expression of CD3D or CD3G and CD3E for unstimulated and stimulated samples respectively. Therefore, it is clear that these cells are not CD4+ T-cells and were removed from subsequent analyses. Since both high and low read depths yielded similar results, in terms of sample clustering, for unstimulated and stimulated samples, only the lower read depth datasets are presented to ensure future relevancy.

As expected from the limited cluster separation on the tSNE plot (Fig. 1), expression signatures were less well defined for the unstimulated sample (Supplementary Fig. 2). However, it was still possible to identify features within clusters and infer cell type identity. For example, clusters representing naïve, cytotoxic, memory, low activity and activated T-cells were identified (Fig. 1a & Supplementary Fig. 3). In contrast, the improved cluster separation for the stimulated sample aided identification as clusters represented more homogenous cell populations and expression differences between clusters were more pronounced. Using a combination of differentially expressed markers and the expression of selected canonical markers for CD4+ T-cell subsets, for example CCR4 (Th2) and CCR6 (Th17), it was therefore possible to assign T-cell subtypes to each cluster, including naïve, memory, early TCR response, regulatory T-cells (Treg), two cytotoxic T-helper subtypes (Th0 & Th17) and various effector cell subsets (Th1, Th2 & Th17) (Fig. 1b & Supplementary Figs. 4–7).

### CCA alignment allows investigation into the cluster specific effect of stimulation

To assess the effect of T-cell receptor (TCR) stimulation and determine the potential of scRNA-seq to identify T-cell signatures under resting and stimulated conditions, it is necessary to merge samples. However, using a standard analysis approach would yield confusing results as cells cluster predominately by stimulatory condition rather than sub-type (Supplementary Fig. 8a,c). We therefore used Seurat to perform a canonical correlation analysis (CCA) to identify shared correlation structures and then align the data into a conserved low-dimensional space.

After alignment, all clusters contained cells originating from both stimulatory conditions and, with the exception of clusters 1 and 2, were comprised of a similar proportion of cells from each condition (Supplementary Fig. 8b,d–f). Further investigation revealed these clusters to express markers indicating a low activity state and naïve phenotype respectively (Supplementary Fig. 9), although the patterns were largely driven by cells originating from the low active stimulated cluster (80%) and the unstimulated naïve cluster (68%) (Supplementary Fig. 8g,h).

When comparing expression differences directly between stimulated and unstimulated cells, over one thousand markers are upregulated and over five hundred downregulated in response to stimulation (Supplementary Fig. 10 and Supplementary Tables 4, 5). These markers included both those traditionally associated with response to stimulation, such as *IL2*, *CD69* and *CCR7*, and less obvious candidates, such as *PIM3* and *MYO1F*. While the change in expression of these markers is reasonably consistent across clusters, some cluster specific effects are observed. For example, cluster 3, representing a persistently naïve cluster, show reduced expression of *CD69*, *TNF*, *LTA*, *IRF4* and *CD40LG* upon stimulation compared to other clusters. Similarly, expression of naïve markers, such as *CCR7*, *GIMAP1* and *GIMAP7* are less downregulated in this cluster and others showing an early or less activated cellular state.

## Discussion

This is the first study to investigate the effect of read depth and cell number for scRNA-seq in CD4+ T-cells under different stimulatory conditions. We found that, first, it was possible to accurately define cell clusters in unstimulated cells at read depths of approximately 60–70 thousand reads per cell; second, stimulated cells showed a higher classification accuracy and finally, cell number had a much more profound effect, requiring a minimum of approximately 2500 cells for accurate classification. These findings are important as they show that significant cost savings can be made by reducing read depth, without affecting the accuracy or relevancy of experimental findings.

The effect of sequencing read depth and cell numbers have previously been studied for single cell RNA-seq^16,17^. However, these studies have either been based on different library preparation methods (e.g. Smart-seq) or focused on classifying more distinct cell populations. For example, previous work in PBMCs showed that classification accuracy remained high, even at read depths as low as 2500 reads per cell^17^. Our findings show that it is not possible to maintain accuracy at such low read depths for CD4+ T-cells, particularly for unstimulated cells. While CD4+ T-cells are a heterogeneous population of multiple, functionally distinct sub-types, their overall similarity is higher compared to other hematopoietic cell lineages and this similarity is even greater in unstimulated cells. Indeed, much higher misclassification was observed for the unstimulated sample even at read depths approaching the full dataset. Reducing cell number also had a greater effect compared to PBMCs illustrating that, while all cell types will be affected by a reduction in either read depth or cell number, the magnitude varies and must be ascertained in individual cell subtypes to ensure optimal study design.

Misclassification of cells occurred mainly for cells on the peripheries of clusters and therefore these cells may represent a transitioning state rather than a distinct cell type. This finding was also observed by Szabo et al*.* using T-cells from lungs, lymph nodes, bone marrow and blood, where the most separation was observed between CD4+ and CD8+ lineages and little separation between neighbouring clusters^18^. Cluster assignment and classification accuracy could be improved, therefore, by removing cells which are furthest away from the cluster centres. However, care must be taken to avoid biasing sub-type frequencies in comparative studies or removing novel, potentially biologically relevant cell states.

Overall, clusters were more distinct in the stimulated sample and it was easier to assign identity to clusters at all sampled read depths compared to the unstimulated sample. Some cell sub-types were common between stimulatory conditions, prior to alignment. For example, both samples contained clusters exhibiting a naïve phenotype (CCR7^+^CD62L^high^CD40L^-^) and a cytotoxic phenotype, although present at different frequencies. For example, as perhaps expected, naïve cells represented 42% of the unstimulated sample, but only 13% of the stimulated sample. The presence of a cluster expressing both cytotoxic markers and Th17-like markers was unexpected, although it has been reported that CRTAM^+^ cytotoxic T-cells can differentiate into Th1- or Th2-like cells and retain their cytotoxic activity^19^. This cluster may therefore represent a Th17-like cytotoxic T-cell subset, although confirmation in more samples is required.

This suggests that to fully characterise cell sub-types in such a homogenous population, such as CD4+ T-cells, some stimulation may be beneficial. While this improves the separation of cell sub-types, it also has a profound impact on the cell, leading to expression of several key effector molecules. This has the potential to alter the cell phenotype and even invoke cell differentiation, skewing the frequencies of the various sub-types. This is evident from the shift of unstimulated naïve cells into other sub-types in the aligned sample and therefore whether to stimulate cells requires careful consideration, based on the experimental aim. Further work is needed to confirm whether sub-type frequencies remain stable after stimulation. To prevent the need for artificial stimulation, and its potential effects, it may be possible to align unstimulated samples to a stimulated reference dataset to allow the successful annotation of cell sub-types. However, this would probably limit the identification of novel, potentially pathogenic sub-types, not present in the reference dataset. Further work would be required to assess the utility of this approach.

An alternative, more robust approach, would be to integrate extensions to scRNA-seq, such as the simultaneous measurement of cell surface proteins and transcriptome (CITE-seq^20^ and REAP-seq^21^), which may help to separate CD4+ T-cells by functional sub-type without the need for stimulation. For example, while candidate marker gene expression, such as CCR4 and CCR6, were used to annotate clusters, some markers did not perform well at distinguishing between sub-types, particularly in the unstimulated sample. The added measurement of cell surface proteins could help improve the discriminatory ability when transcript expression is low. Furthermore, by pooling both conditions together, for example by using cell hashing^22^, it would mitigate batch effects and allow a direct comparison between unstimulated and stimulated cells. While this is unlikely to align cells entirely, it would be advantageous for accurately comparing between cell states.

Despite the strong effect of stimulation, after CCA alignment, cells did indeed cluster independent of stimulatory status and shared cell types could be identified, allowing the direct comparison between cells with very different activities and expression profiles. This indicates that it should be possible to compare similar samples with different disease activity, efficiently controlling for any confounding caused, but confirmation will require testing patient samples with a spectrum of disease activities.

By producing a high depth, high cell number primary CD4+ T-cell scRNA-seq dataset, both with and without TCR stimulation, we have shown that while the accuracy of cell type classification in scRNA-seq is effected by both sequencing read depth and cell number, cell number has a much greater effect. The study provides an important, initial indication of both sequencing and cell number requirements for scRNA-seq experiments using CD4+ T-cells. Our data shows that for CD4+ T-cells, 60–70 thousand reads per cell should be sufficient, providing cell number is maintained, and supports droplet-based scRNA-seq as the preferred platform due to the high cell numbers attainable. While it is unexpected that inter-individual variability would significantly change this recommendation, it should be noted that it is based on a single individual and as such, further validation would be required to support this conclusion further. Our CCA analysis shows that by aligning samples using shared structures we were able to identify known cell subtypes and characterise the transcriptome of these subtypes, as well as their response to stimulation. Again, while the effect of stimulation has been shown to be similar between samples in bulk RNA-seq^23^, increased sample numbers would be required to confirm this in scRNA-seq. Despite this, these findings suggest it is possible to directly compare samples with different disease activities, an essential step in studies investigating treatment response.

## Methods

### Ethical approval and consent to participate

Informed consent from participates was taken by the NHS Blood and Transplant (NHSBT) service and samples provided were covered under ethical approval by the North West Multi-Centre Research Ethics Committee (MREC 99/8/84). All experiments were performed in accordance with relevant guidelines and regulations.

### Sample preparation

Peripheral blood mononuclear cells (PBMCs) were extracted from a leukocyte cone collected from a healthy human volunteer from the NHSBT service by ficoll density gradient centrifugation. CD4+ T-cells were then isolated from 100 million PBMCs using the EasySep Human CD4+ T Cell Isolation Kit (STEMCELL Technologies, catalog # 17952) using negative selection following the manufacture’s protocol. Flow cytometry analysis ensured a high purity (97.5%) CD4+ T-cell population was obtained (Supplementary Fig. 1a). Cells were seeded at 1 million cells/ml in RPMI, 10% FBS, pen/strep and incubated overnight at 37˚C. Half were stimulated using Dynabeads Human T-Activator CD3/CD28 beads (ThermoFisher Scientific, Cat. No. 111.31D) for 4 h at a ratio of 1:1 (beads:cells); the remaining half were kept at the same conditions with no stimulation. After stimulation, a magnet was used to collect the Dynabeads to ensure they did not interfere with downstream processing. Successful stimulation was confirmed by flow cytometry analysis (Supplementary Fig. 1b).

### Single cell RNA-seq

Single cell RNA-seq libraries were prepared using the Chromium Single Cell Controller (10x Genomics, Pleasanton, CA) using the Chromium Single Cell 3′ Library & Gel Bead v2 kit. Briefly, cell suspensions were diluted in nuclease-free water according to manufacturer instructions to obtain a target cell recovery of 6000 cells (Supplementary Table 1). Remaining steps were carried out according to the manufacturers’ instructions using 12 cycles for cDNA amplification and 14 cycles for the sample index PCR.

Final libraries were sequenced on one flow cell of an Illumina HiSeq 4000 (Illumina, San Diego) with a read length of 26 bp for read 1 (cell barcode and unique molecule identifier (UMI)), 8 bp i7 index read (sample barcode), and 98 bp for read 2 (RNA read) to yield approximately 1.25 billion reads per sample (208,000 reads per cell).

### Data processing and quality control

Raw sequence reads were aligned against the human reference (GENCODE Human Release 26 (GRCh38.p10)) using the Cell Ranger 2.1.0 pipeline (10x Genomics) and processed further using Seurat—R toolkit for single cell genomics 2.3.0^24^. Initial filtering removed cells expressing less than 200 genes and genes that were expressed in less than 3 cells. Poor quality cells and potential multiplets were classified as outside three median absolute deviations (MADs) for percentage mitochrondrial content, number of genes and number of UMIs and removed.

### Read depth subsampling

To analyse the effect of read depth, raw sequence reads were subsampled to varying target depths using two methods. The first approach simulated biological variability by exploiting discrete sequencing lanes. By simply taking all combinations of 1, 2 or 3 lanes, sampling of intended read depths of approximately 50 K, 100 K and 150 K mean reads per cell was performed, followed by replication (Supplementary Table 2). An additional method to subsample reads allowing lower and finer control of target read depths of between 20 K-100 K mean reads per cell at 10 K intervals was also undertaken. Firstly, raw reads were converted to unmapped BAM format and merged using Picard Tools 2.9.2 and Samtools 1.7 respectively. Samtools was then used to randomly subsample reads and convert back to FASTQ format. Subsampling at each depth was replicated three times and processed using the Cell Ranger pipeline as the full datasets.

### Cell number subsampling

To investigate the impact of the number of cells in a sample, cells passing quality control at selected read depths, including the high sequencing depth datasets, were randomly subsampled to obtain 500, 1000, 1500, 2000, 2500, 3000, 3500 and 4000 cells (Supplementary Table 3) from the Seurat object using R 3.4.2. Each subsampled dataset was filtered and poor quality cells and potential multiplets were removed using the same approach as the full datasets.

### Clustering of cells

Following quality control, gene expression counts were normalised using the log normalisation method implemented in Seurat using default parameters. Highly variable genes were identified for downstream analysis using Seurat’s *FindVariableGenes* function using an average expression cut-offs of 0.0125 and 8 and a lower dispersion cut-off of 0.5. Data was scaled and centred, regressing out number of UMIs and percentage mitochondrial content. Principle component analysis was performed using the highly variable genes to identify the top 20 principle components (PCs). Graph based clustering of cells was performed using Seurat’s *FindClusters* method using a resolution of 1 and selected PCs. PCs were selected manually based on visual inspection of PC heat maps on the 500 most ‘extreme’ cells. Clustering was visualised using t-distributed stochastic neighbour embedding (tSNE) plots based on the same number of PCs. Manual inspection of the clusters identified for each high depth dataset showed two clusters in each condition, which showed minimal differences. These clusters were therefore merged prior to comparing clusters.

### Cell type classification accuracy

To assess the accuracy of the graph based clustering on cell type classification, each subsampled dataset was compared against the relevant high sequencing depth dataset. For the subsampled cell number datasets, this corresponded to all cells identified at that particular read depth. To relate clusters identified in the subsampled datasets to clusters identified in the high depth dataset, a random forest classifier was trained on the relevant high depth dataset based on the highly variable genes. This classifier was then used to assign a predicted identity to each cell in the subsampled dataset. The identity of each cluster was then selected based on the highest proportion of cells in the subsampled dataset. For example, if 90% of cells within a cluster in the subsampled dataset were predicted to match cluster 2 in the high depth dataset, the entire subsampled cluster would be assigned to cluster 2. The approach is therefore unbiased and is not reliant on specifying a particular cell type, or selecting a list of representative genes for that cell type. Confusion matrices were then calculated and accuracy determined using the caret package in R.

### Cluster cell type identification

Cluster cell types were annotated using a combination of differentially expressed markers, identified using the Seurat FindAllMarkers and FindMarkers functions, and the expression of selected canonical markers for CD4+ T-cell subsets, for example *CCR4* (Th2) and *CCR6* (Th17).

### Comparison between stimulatory conditions

To compare samples before and after TCR stimulation, we used the Seurat canonical correlation analysis (CCA) and alignment strategy. To identify shared correlation structures across both datasets, we merged samples and ran the CCA on the union of the top 2000 most variable genes from each sample (3487 genes). Cells showing poor correlation between CCA and principle component analysis (PCA) were removed (> twofold difference) and the remaining cells aligned using the ‘AlignSubspace’ command on the first 25 CCA components. Cells were clustered as previously described using the first 25 aligned CCA components and visualised using tSNE plots as before.

## Supplementary information

Supplementary Information.

Supplementary Tables 4 and 5.

## Data Availability

All data generated during this study is available through the Gene Expression Omnibus accession GSE147928.
