# Supplementary material for: Characterisation of CD4+ T-cell subtypes using single cell RNA sequencing and the impact of cell number and sequencing depth
Source: Sci Rep. 2020 Nov 13;10:19825. doi: 10.1038/s41598-020-76972-9 (PMC7666207; doi:10.1038/s41598-020-76972-9)
Supplement: Supplementary file 1 — Supplementary Information. [file 41598_2020_76972_MOESM1_ESM.pdf]

Characterisation of CD4+ T-cell subtypes using single cell RNA sequencing and the impact of cell number and sequencing depth

James Ding[1], Samantha L Smith[1], Gisela Orozco[1], Anne Barton[1,3], Steve Eyre[1] & Paul Martin[1,2,*]

1. Centre for Genetics and Genomics Versus Arthritis, Centre for Musculoskeletal Research, Manchester Academic Health Science Centre, University of Manchester, Manchester, UK

2. The Lydia Becker Institute of Immunology and Inflammation, Faculty of Biology, Medicine and Health, University of Manchester, Manchester, UK

3. NIHR Manchester Biomedical Research Centre, Manchester University NHS Foundation Trust, Manchester Academic Health Science Centre, Manchester, UK

* Correspondence to: Paul Martin. Centre for Genetics and Genomics Versus Arthritis. The University of Manchester. AV Hill Building, Oxford Road. Manchester M13 9PT UK paul.martin-2@manchester.ac.uk +44(0)161 306 8507

Supplementary Table 1 Concentration and number of cells loaded by sample.

**Sample**	**Cell concentration (cells/µl)**	**Number of cells loaded**
**Stimulated**	1,700	10,370
**Unstimulated**	1,600	10,400

Supplementary Table 2 Sequencing lanes selected for each replicate and intended read depth per cell.

	**Lane 1**	**Lane 2**	**Lane 3**	**Lane 4**	**Intended Reads per Cell**
**1 lane rep 1**	✓				50,000
**1 lane rep 2**		✓			50,000
**1 lane rep 3**			✓		50,000
**1 lane rep 4**				✓	50,000
**2 lanes rep 1**	✓	✓			100,000
**2 lanes rep 2**			✓	✓	100,000
**2 lanes rep 3**		✓	✓		100,000
**2 lanes rep 4**	✓		✓		100,000
**2 lanes rep 5**	✓			✓	100,000
**2 lanes rep 6**		✓		✓	100,000
**3 lanes rep 1**	✓	✓	✓		150,000
**3 lanes rep 2**	✓	✓		✓	150,000
**3 lanes rep 3**	✓		✓	✓	150,000
**3 lanes rep 4**		✓	✓	✓	150,000

Supplementary Table 3 Subsampled read depths selected for cell number subsampling.

**Sample**	**Approximate Reads per Cell**							
	**50,000**	**65,000**	**75,000**	**80,000**	**100,000**	**165,000**	**200,000**	**250,000**
**Stimulated**								
**Unstimulated**								

Supplementary Table 4 Differentially expressed upregulated genes between stimulated and unstimulated samples by merged cluster identity.

Supplementary Table 5 Differentially expressed downregulated genes between stimulated and unstimulated samples by merged cluster identity.

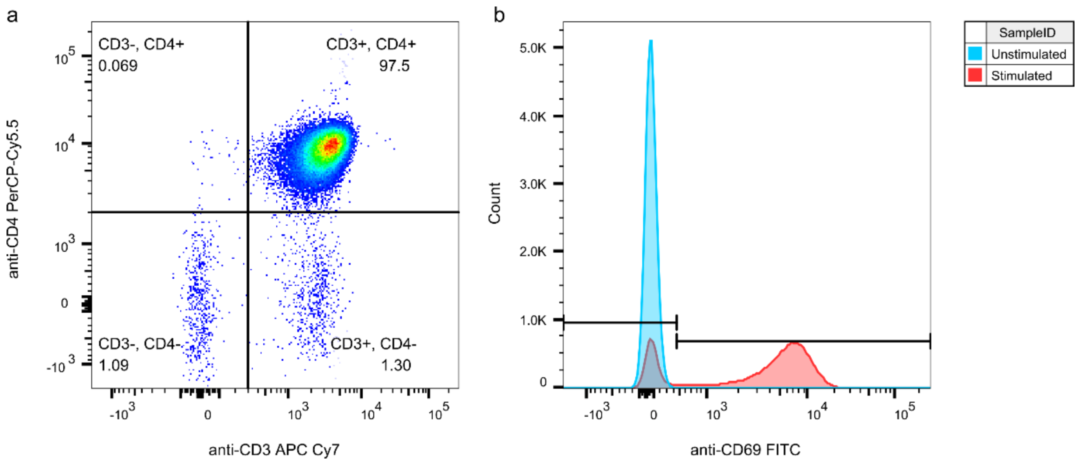

Supplementary Figure 1 Flow cytometry data showing (a) sample purity and (b) confirmation of stimulation.

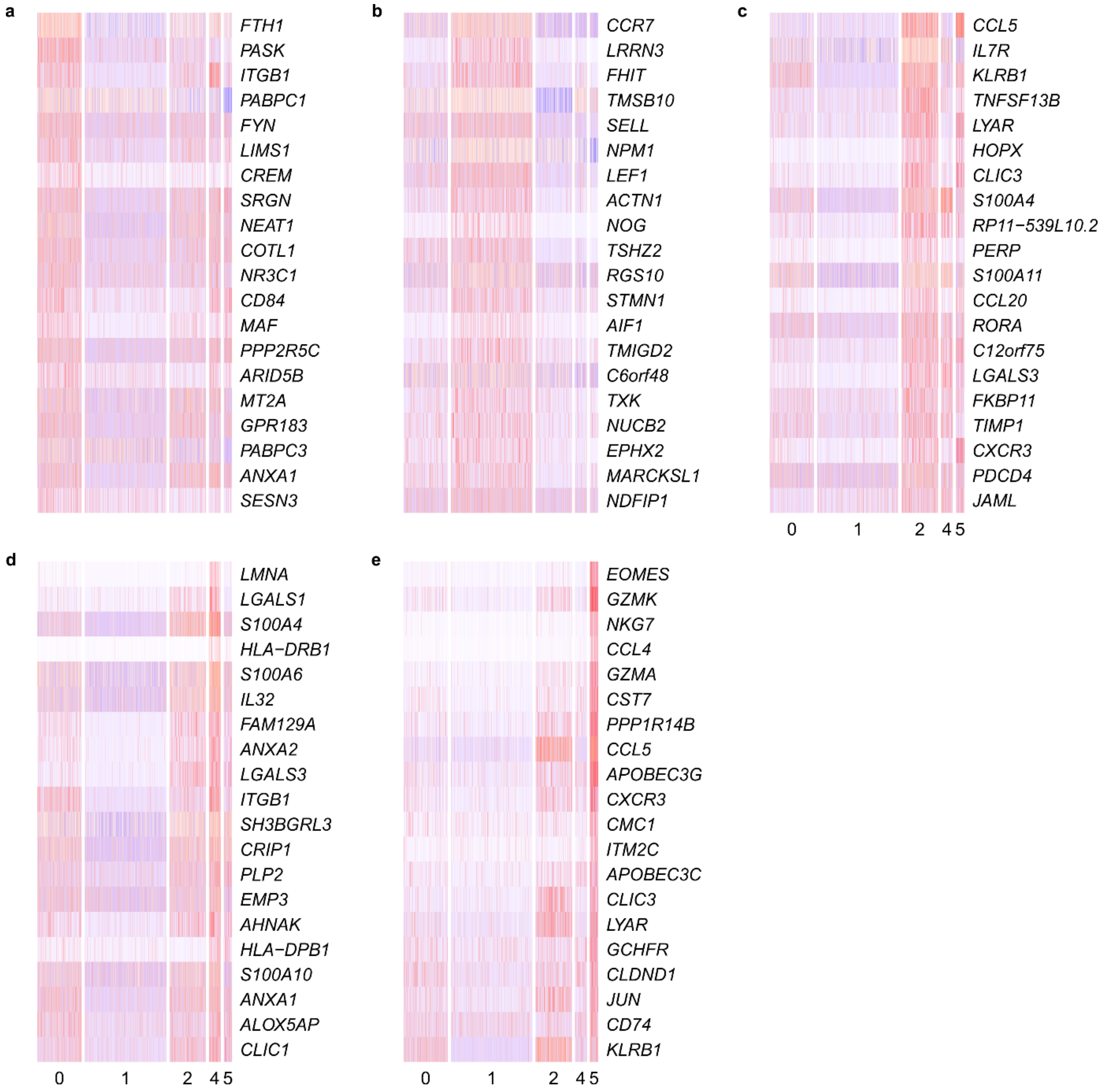

Supplementary Figure 2 Heatmap of the top 20 differentially expressed markers for the unstimulated sample by cluster. Markers differentially expressed in each cluster are labelled a-e sequentially.

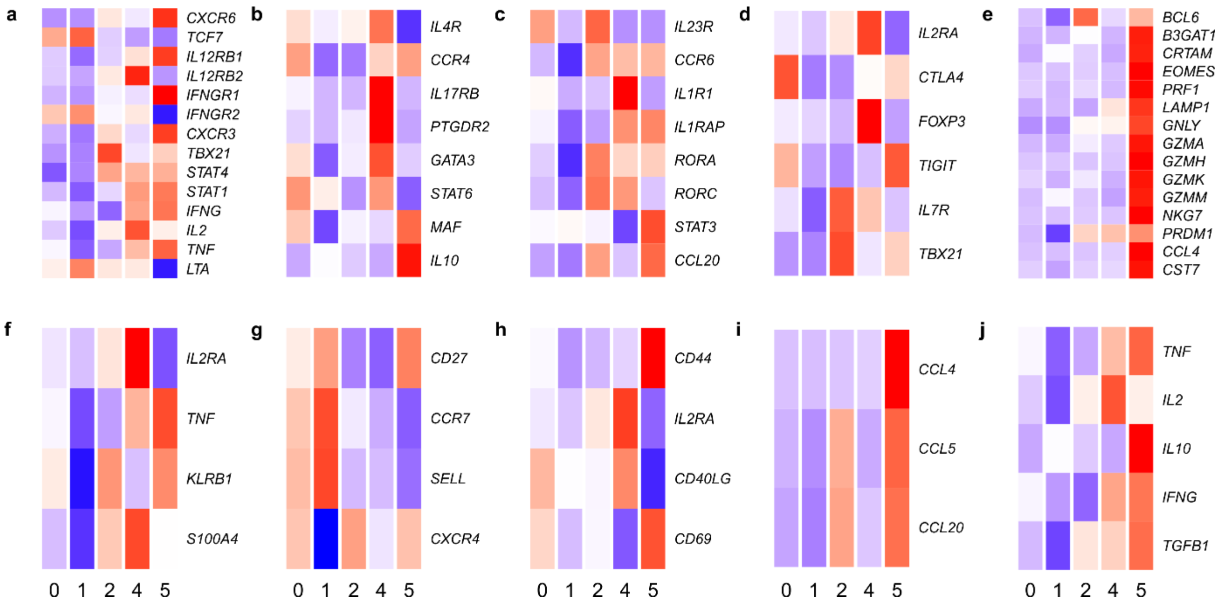

Supplementary Figure 3 Heatmap of selected markers indicative of various CD4+ T-cell sub-types and activation states for the unstimulated sample. Panels a-d are markers expressed in (a) Th1, (b) Th2, (c) Th17 and (d) regulatory CD4+ T-cells. Panels e-j show expression of (e) cytotoxicity markers, (f) memory markers, (g) naïve markers, (h) activation markers (i) chemokines and (j) cytokines.

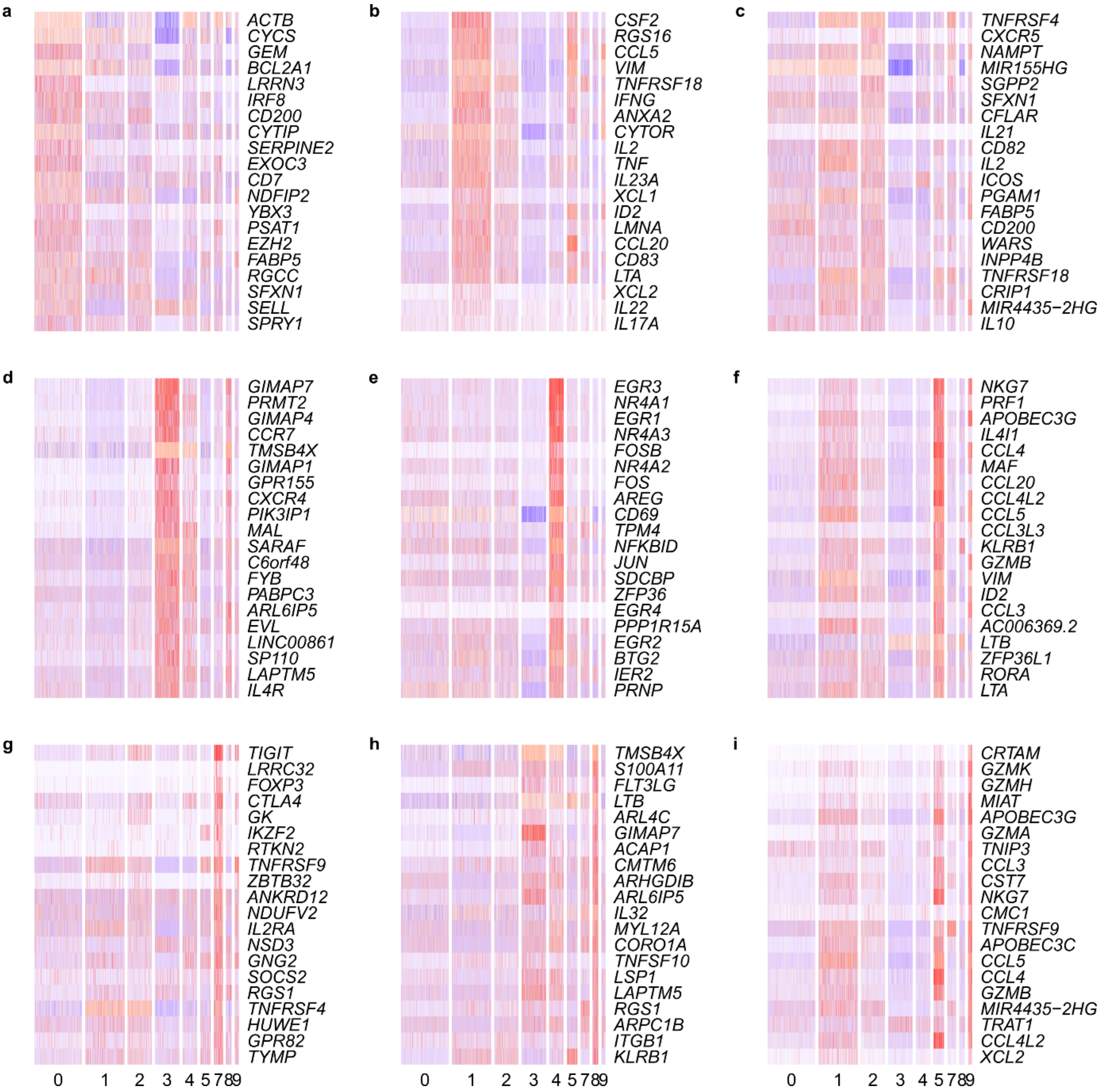

Supplementary Figure 4 Heatmap of the top 20 differentially expressed markers for the stimulated sample by cluster. Markers differentially expressed in each cluster are labelled a-i sequentially.

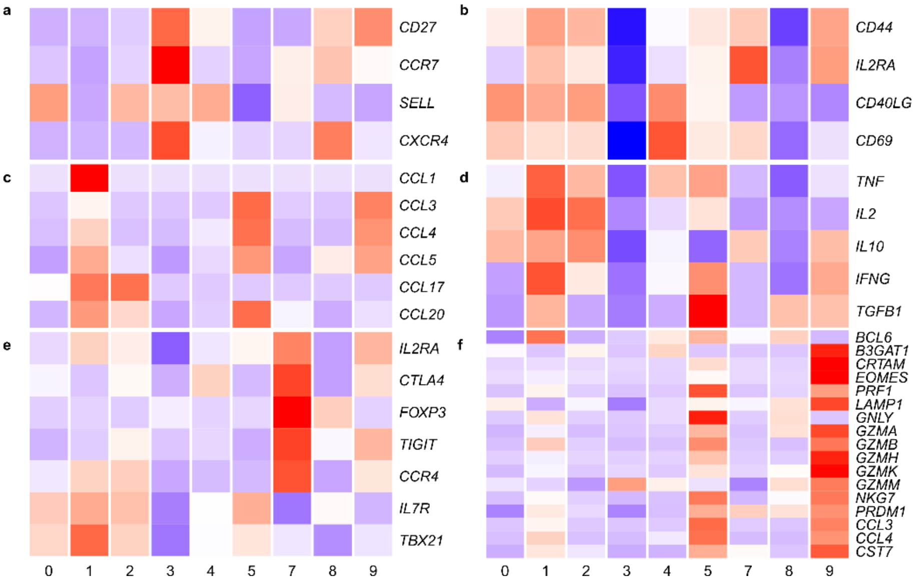

Supplementary Figure 5 Heatmap of selected markers for the stimulated sample. Markers are shown to indicate (a) naïve T-cells, (b) activation, (c) chemokine expression, (d) cytokine expression, (e) regulatory T-cells and (f) cytotoxicity.

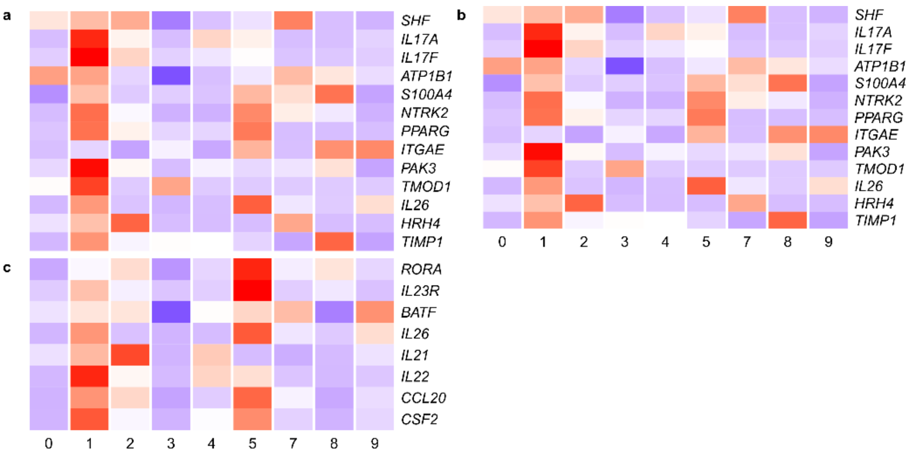

Supplementary Figure 6 Heatmap of selected Th17 markers for the stimulated sample. Panels a-c are (a) core Th17 markers, (b) Th17.1 markers and (c) additional Th17 markers.

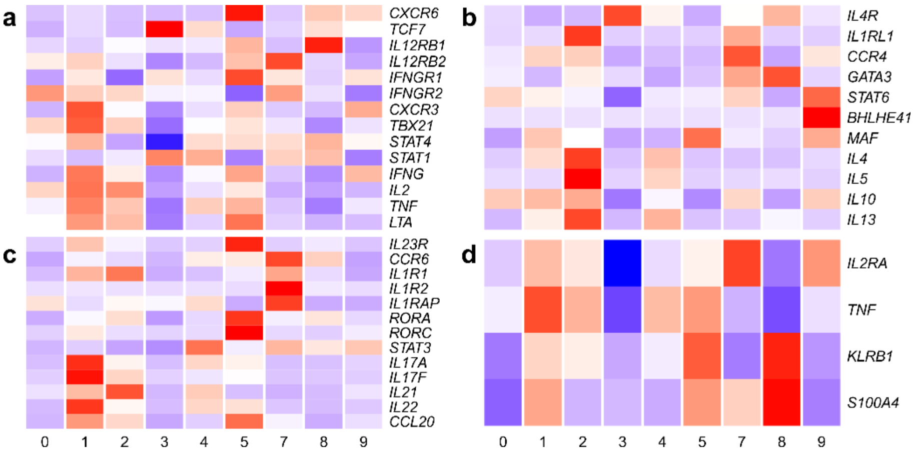

Supplementary Figure 7 Heatmap of selected T helper subset markers for the stimulated sample. Panels a-d are markers expressed in (a) Th1, (b) Th2, (c) Th17 and (d) memory CD4+ T-cells.

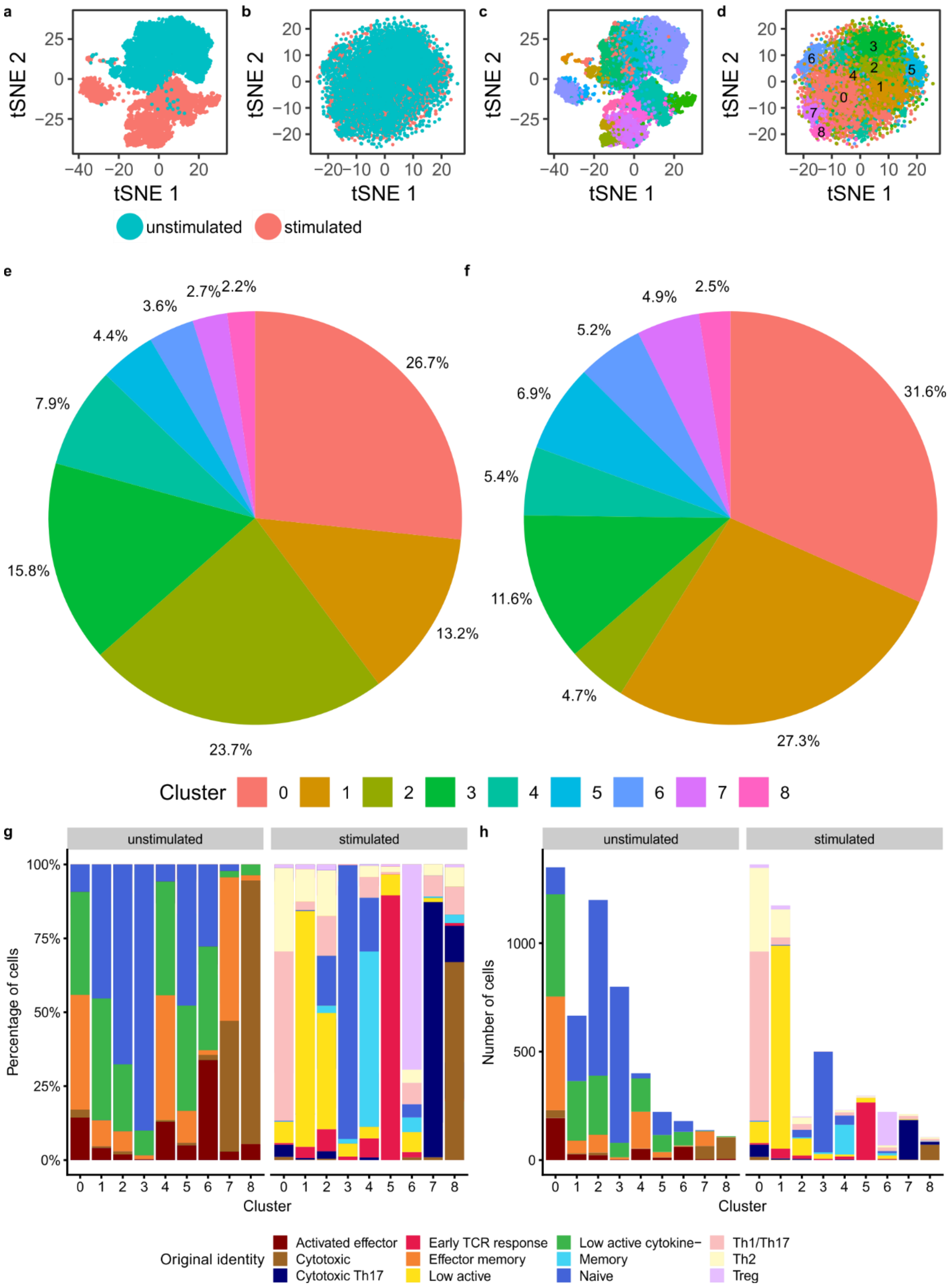

Supplementary Figure 8 Comparison between stimulatory conditions after merging. Panels a-d show tSNE plots of (a) merging without canonical correlation analysis (CCA) by stimulatory condition, (b) merging with CCA by stimulatory condition, (c) merging without canonical correlation analysis (CCA) by cluster identity and (d) cluster identity after merging with CCA. Panels e and f show the percentage of (e) unstimulated and (f) stimulated cells contributing to each merged cluster. Panels g and h show the (g) percentage and (h) cell numbers contributing to each merged cluster by original cluster identity (Figure 1).

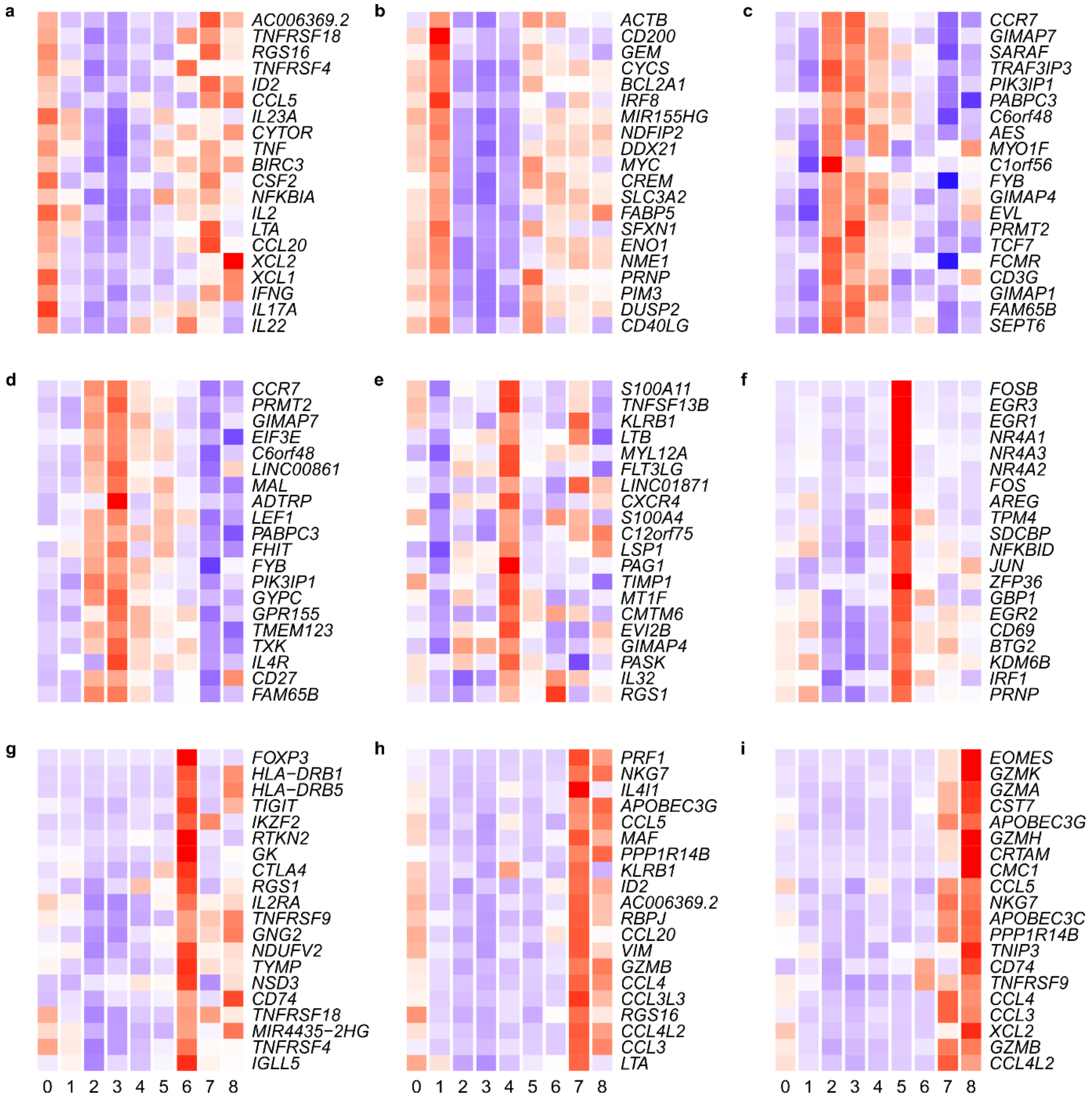

Supplementary Figure 9 Heatmap of the top 20 differentially expressed markers for the merged sample by cluster. Markers differentially expressed in each cluster are labelled a-i sequentially.

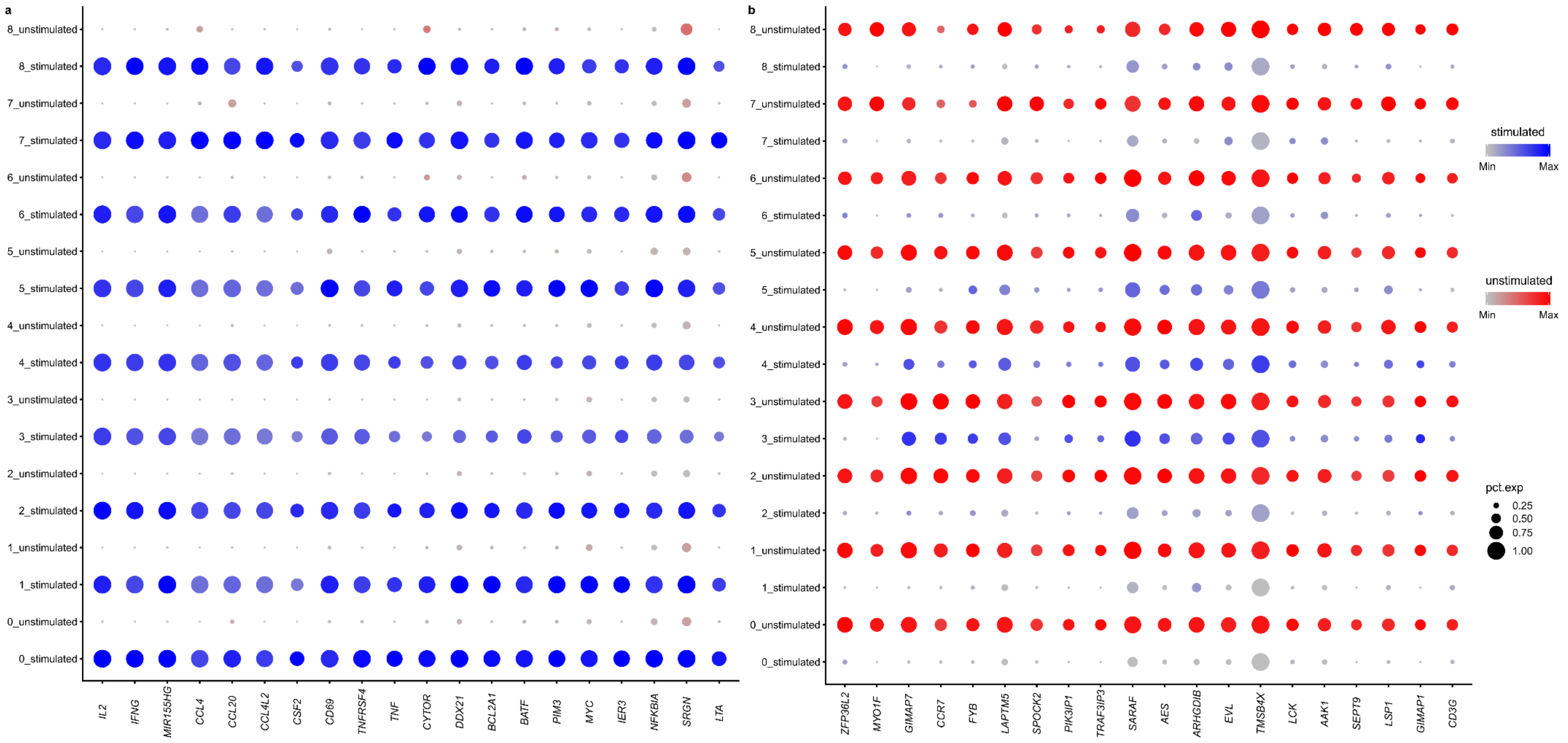

Supplementary Figure 10 Top 20 differentially expressed (a) upregulated and (b) downregulated genes between stimulated and unstimulated samples by merged cluster identity.
